# Antioxidant Mechanism of* Rutin* on Hypoxia-Induced Pulmonary Arterial Cell Proliferation

**DOI:** 10.3390/molecules191119036

**Published:** 2014-11-18

**Authors:** Qian Li, Yanli Qiu, Min Mao, Jinying Lv, Lixin Zhang, Shuzhen Li, Xia Li, Xiaodong Zheng

**Affiliations:** 1 Department of Pharmaceutical Analysis, College of Pharmacy, Harbin Medical University, Nangang District, Harbin 150081, China; E-Mails: liqian@ems.hrbmu.edu.cn (Q.L.); yingzi1314179@126.com (Y.Q.); m15645109321@163.com (J.L.); fengyucaihong78@sohu.com (X.L.); 2 Bio-pharmaceutical Key Laboratory of Harbin, Harbin Medical University, Harbin 150081, China; E-Mails: mmarine510704@hotmail.com (M.M.); muzqian@sina.com (L.Z.); yw198269@163.com (S.L.); 3 Department of Pathophysiology, Harbin Medical University-Daqing, Daqing 163319, China

**Keywords:** *rutin*, reactive oxygen species (ROS), NADPH oxidase 4, proliferation, hypoxia

## Abstract

Reactive oxygen species (ROS) are involved in the pathologic process of pulmonary arterial hypertension as either mediators or inducers. *Rutin* is a type of flavonoid which exhibits significant scavenging properties on oxygen radicals both* in vitro* and* in vivo*. In this study, we proposed that *rutin* attenuated hypoxia-induced pulmonary artery smooth muscle cell (PASMC) proliferation by scavenging ROS. Immunofluorescence data showed that *rutin* decreased the production of ROS, which was mainly generated through mitochondria and NADPH oxidase 4 (Nox4) in pulmonary artery endothelial cells (PAECs). Western blot results provided further evidence on *rutin* increasing expression of Nox4 and hypoxia-inducible factor-1α (HIF-1α). Moreover, cell cycle analysis by flow cytometry indicated that proliferation of PASMCs triggered by hypoxia was also repressed by *rutin*. However, *N*-acetyl-L-cysteine (NAC), a scavenger of ROS, abolished or diminished the capability of *rutin* in repressing hypoxia-induced cell proliferation. These data suggest that *rutin* shows a potential benefit against the development of hypoxic pulmonary arterial hypertension by inhibiting ROS, subsequently preventing hypoxia-induced PASMC proliferation.

## 1. Introduction

Hypoxic pulmonary arterial hypertension (HPH) is a progressive disorder characterized by endothelial dysfunction, smooth muscle proliferation, intimal and/or medial layer remodeling, and right ventricular failure [1,2]. Remodeling of pulmonary blood vessels results from the proliferation of pulmonary arterial smooth muscle cells (PASMCs) and pulmonary arterial endothelial cells (PAECs) migration [3,4]. Endothelial cells are generally recognized as the main regulators of vascular function. Imbalance between vasoconstriction and vasodilation is characterized by ECs dysfunction [5]. PAECs dysfunction is involved in the development of HPH by contribution to the disturbed migration and proliferation of PASMCs [6]. However, the exact causes of HPH are still under investigation.

It has been shown that increasing oxidative stress augments HPH [7], whereas reducing oxidative stress reverses HPH [8]. These data suggest oxidative stress is implicated in the development of HPH [9]. During hypoxic condition, reactive oxygen species (ROS) released from PAECs, subsequently diffuses to PASMCs and causes calcium influx, which leads to pulmonary artery constriction [10]. Furthermore, ROS, such as superoxide anions and hydrogen peroxide (H_2_O_2_) serve as regulators in the vascular smooth muscle cells proliferation [11,12]. NADPH oxidase 4 (Nox4) is a major isoform of NADPH oxidase responsible for the production of ROS which functions as a signaling molecule in cell survival and normal function [13].

Several signal transduction pathways and transcription factors are activated that could be involved in PASMC proliferation [14,15]. Proliferating cell nuclear antigen (PCNA) plays an important role in nucleic acid metabolism acting as a component for repair and replication machinery, and is known as a molecular marker for proliferation [16,17]. A previous study by our group indicated PCNA expression serves as an indicator for PASMCs proliferation [18]. Animal studies revealed a role for hypoxia-inducible factor-1 (HIF-1) in the development of HPH [19,20]. The β subunit of HIF-1 (HIF-1β) is constitutively expressed, whereas the α subunit (HIF-1α) is typically not detectable under normoxic conditions. Moreover, accumulation of HIF-1α protein was observed in the lungs of PAH patients. A molecule that targets HIF-1 may provide a therapeutic potential for HPH.

**Figure 1:**
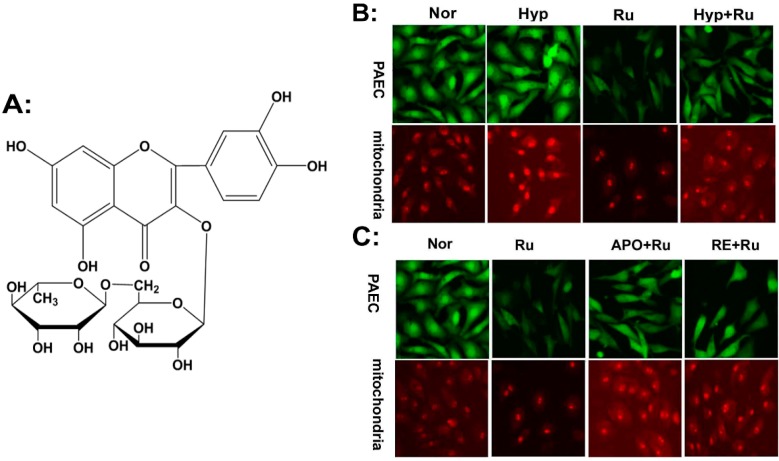
Immunofluorescence staining using CM-H_2_DCFDA or mitosox to evaluate the production of reactive oxygen species (ROS) in pulmonary artery endothelial cells (PAECs) and mitochondria. (**A**) Chemical structure of *rutin*. (**B**) The increased production of ROS in cells (Green) and mitochondria (Red) induced by hypoxia was reduced when treated with *rutin*. (**C**) Cells were pretreated with APO (apocynin, NADPH oxidases inhibitor), RE (rotenone, mitochondria inhibitor), showed the reverse effect of *rutin* on pulmonary endothelial cells, respectively. ROS was assessed by a laser-scanning confocal microscope after treatment by CM-H_2_DCFDA for the detection of the whole cells (Green) and mitosox (Red) for the detection of mitochondria respectively. Scale bars were 100 μm. Nor indicates normoxia, Hyp indicates hypoxia, Ru: *rutin*.

*Rutin*, 3-((6-*O*-(6-deoxy-α-l-mannopyranosyl)-β-d-glucopyranosyl)oxy)-2-(3,4-dihy-droxyphenyl)-5,7-dihydroxy-4H-1-benzopyran-4-one (shown in Figure 1A), is the glycoside between the flavonol quercetin and the disaccharide rutinoside. *Rutin* restrains the oxidative progress. Based on its significant scavenging properties on oxidizing species, *rutin* has been implemented in ROS-related diseases, such as gastric lesions and diabetes [21,22]. Besides, *rutin* demonstrates several pharmacological activities, including anti-inflammatory, anti-allergic and vasoactive properties. In a previous study, we showed *rutin* attenuated hypoxic pulmonary vasoconstriction [23]. However, whether *rutin* has an effect on the abnormal proliferation in PASMCs is still unclear. In this study, we examined the effect of *rutin* on (1) scavenged PAECs ROS production; mitochondrial Nox4, which is connected to ROS production [24]; (2) inhibition of the expression of PCNA; and (3) decreased HIF-1α expression, which was activated as a result of cell adaptive response under hypoxia. The aim was to determine whether *rutin* has an adjuvant therapy effect on HPH.

## 2. Results and Discussion

### 2.1. Rutin Abolished Mitochondrial and Nox4-Generated ROS Production

The production of ROS was measured with CM-H_2_DCFDA. Hypoxia increased the ROS production in PAECs, as indicated by the increased fluorescence intensity (Figure 1B; Green). Mitochondria serve as the main organelle of cell metabolism and as an important producer of ROS. Then we examined ROS production in mitochondria using mitosox. The enhanced red flurorescence intensity indicated hypoxia also increased the ROS production in mitochondria (Figure 1B; Red). As proposed, *rutin* abolished the abnormal rise of ROS triggered by hypoxia both in PAECs and mitochondria (Figure 1B). Surprisingly, pre-treatment with APO (apocynin, NADPH oxidases inhibitor) or RE (rotenone, Mitochondria inhibitor) attenuated *rutin*’s effect of decreasing ROS production (Figure 1C). The results demonstrated that *rutin* abolished the production of ROS, generated through Nox4 and mitochondrial under hypoxic conditions in PAECs.

### 2.2. Rutin Influenced the Expression of Nox4 under Hypoxia

**Figure 2:**
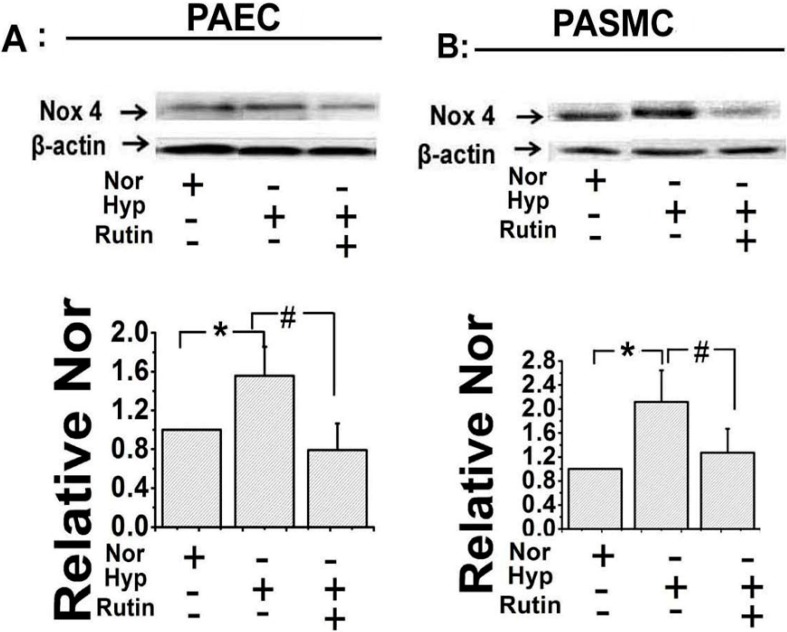
The regulative effect of *rutin* on the expression of NADPH oxidase 4 (Nox4) under hypoxia was evaluated by Western Blot. Under hypoxia, the activity of Nox4 was decreased when treated with *rutin*, or *N*-acetyl-l-cysteine (NAC) (5 μmol/L) suppressed the effect of *rutin* evidently in PAECs (**A**) and pulmonary arterial smooth muscle cells (PASMCs). (**B**) All of the values were denoted as mean ± SD; * *p* < 0.05, *versus* normal; ^#^ *p* < 0.05, *versus* Hyp. Nor indicates normoxia, Hyp indicates hypoxia.

The decreased ROS production could be due to two factors, one less is produced, two more is scavenged. In order to examine which way was involved for the effect of *rutin*, the expression of Nox4 was elevated under hypoxic circumstances. Western blotting showed hypoxia increased Nox4 expression, and *rutin* decreased the up-regulation of Nox4 induced by hypoxia in both the PAECs (Figure 2A) and the PASMCs (Figure 2B). These data indicated *rutin* diminished ROS production by affecting Nox4 activity.

### 2.3. Rutin Inhibited Hypoxia-Stimulated PAECs Migration and PASMCs Proliferation

**Figure 3:**
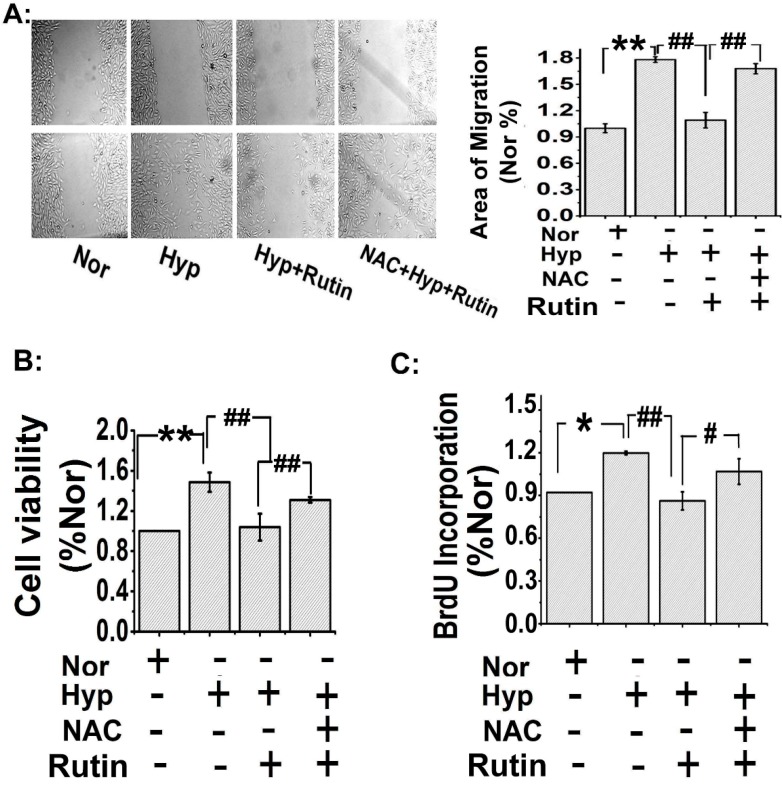
*Rutin* blocked PAECs migration and PASMCs proliferation under hypoxia by regulating ROS. (**A**) The effect of hypoxia (8 h) on cell migration was blocked by *rutin* (*n* = 3), which was attenuated by NAC. Scale bars are 100 μm. (**B**) Cell viability was measured by MTT, the results were consistent with scratch-wound assay,* rutin* abolished hypoxia-increasing PASMCs viability, which was blocked with NAC. (**C**) NAC (ROS scavenger, 5 μmol/L) increased 5-bromodeoxyuridine incorporation compared with the *rutin* group under hypoxia. All of the values were denoted as mean ± SD; * *p* < 0.05, ** *p* < 0.01,* versus* normal; # *p* < 0.05, ^##^ *p* < 0.01* versus* Hyp, *n* = 3. Nor indicates normoxia, Hyp indicates hypoxia.

In order to examine the effect of *rutin* on PAECs migration under hypoxia, we performed a PAECs scratch-wound assay. The results showed that hypoxia significantly promoted PAECs migration, as shown in Figure 3A, *rutin* inhibited the hypoxia-trigged PAECs migration. Consistent with the results in Figure 1C, scavenged ROS by *N*-acetyl-l-cysteine (NAC, 5 μmol/L) abolished the effect of *rutin* on inhibition of PAECs migration.

**Figure 4:**
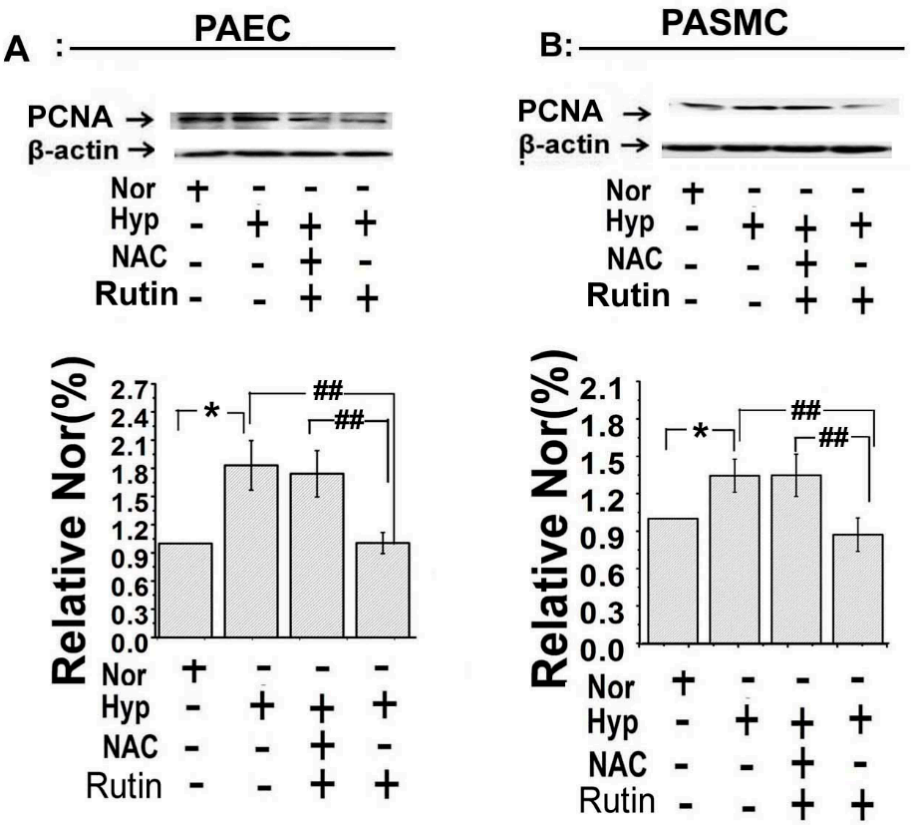
*Rutin* reversed the increased expression of proliferating cell nuclear antigen (PCNA) during hypoxia. Evaluation of the expression of PCNA in PAECs (**A**) and PASMCs (**B**) by Western Blot. The abnormal increased activity of PCNA induced by hypoxia was alleviated by *rutin*. All of the values were denoted as mean ± SD; * *p* < 0.05, *versus* normal; ^##^ *p* < 0.01* versus* Hyp, *n* = 3. Nor indicates normoxia, Hyp indicates hypoxia.

Hypoxia induced PASMCs proliferation is a character of hypoxia induced pulmonary hypertension. So we then examined whether *rutin* protected HPH through inhibiting PASMCs proliferation. Firstly, cell viability was measured by MTT, as shown in Figure 3B, hypoxia increased cell viability, and the effect was inhibited by *rutin*. Consistent with the PAECs migration data, pre-treatment with NAC, diminished the effect of *rutin*. Secondly, 5-bromodeoxyuridine incorporation assay showed a similar result, hypoxia increased 5-bromodeoxyuridine incorporation in PASMCs, the increasing effect was suppressed by *rutin*, whereas it was significantly attenuated in the presence of NAC (Figure 3C). Thirdly, PCNA, a symbol protein of cell proliferation and further remodeling of PA during the pathologic process in PAH was detected [17,25]. Hypoxia significantly increased the expression of PCNA in both the PAECs and the PASMCs (Figure 4). However, *rutin* reduced the hypoxia-induced upregulation expression of PCNA, whereas the inhibition effect was abolished by NAC (Figure 4). These results indicated that *rutin* inhibited hypoxia-induced PAECs migration and PASMCs proliferation, which may be through a ROS-dependent pathway.

### 2.4. Effect of Rutin on Hypoxia-Induced ROS on Cell Cycle Progression and Microtubule Dynamic Stability

**Figure 5:**
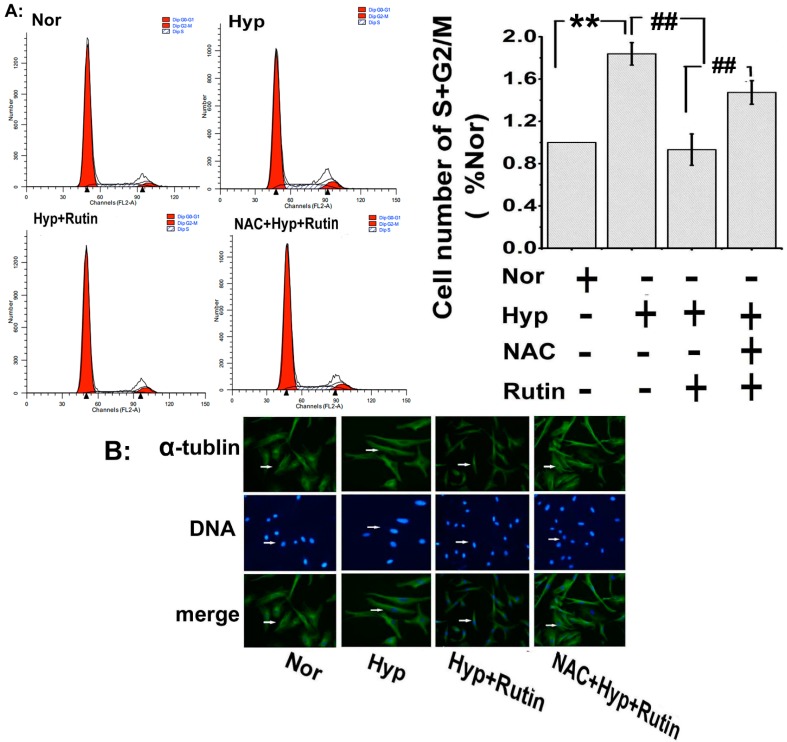
The influence of *rutin* on the activity of PASMCs was detected with flow cytometry and the expression of α-tubulin was recorded with immunohistochemistry. (**A**) Hypoxia promoted PASMCs cycle progression and increased the cell number in S plus G2/M phases, which was suppressed in the presence of *rutin.* The effect of* rutin* on hypoxia-induced enhanced cycle progression was eliminated with scavenged ROS by NAC. (**B**) The expression of α-tubulin in PASMCs was upregulated under hypoxia which was blocked by *rutin*, and the role of *rutin* was eliminated in the presence of NAC (ROS scavenger, 5 μmol/L). Scale bars were 100 μm. All of the values were denoted as mean ± SD; ** *p* < 0.01,* versus* normal; ^##^ *p* < 0.01, *versus* Hyp. *n* = 3. Nor indicates normoxia, Hyp indicates hypoxia.

In order to confirm *rutin* regulated PASMCs proliferation process, we measured the cell cycle progression by flow cytometry, as shown in Figure 5A; hypoxia promoted PASMCs transit into S plus G_2_/M phases, *rutin* was able to repress the cell cycle progression whereas *rutin*’s effects were attenuated with scavenged ROS by NAC (Figure 5A). Moreover, hypoxia induced the expression of housekeeper protein α-tubulin in the nucleus of PASMCs (Figure 5B), which may promote mitotic spindle formation, and then contribute to PASMCs proliferation [26].* Rutin* reversed this increasing expression of α-tubulin, and the reversed effect was attenuated after scavenging ROS with NAC.

### 2.5. The Upregulated Expression of HIF-1α Induced by Hypoxia Was Depressed by Rutin

**Figure 6:**
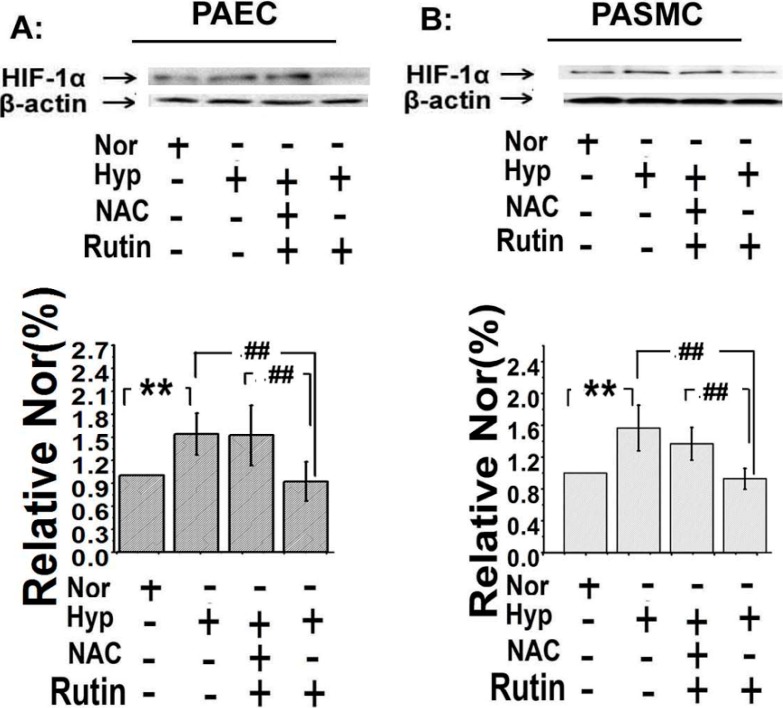
*Rutin* affected the expression of hypoxia-inducible factor-1α (HIF-1α) when exposed under hypoxia. The upregulated expression of HIF-1α of PAECs (**A**) and PASMCs (**B**) induced by hypoxia was repressed by *rutin*, and the effect of* rutin* on hypoxia-induced overexpression of HIF-1α was alleviated by NAC. All of the values were denoted as mean ± SD; ** *p* < 0.01,* versus* normal; ^##^ *p* < 0.01*versus* Hyp. *n* = 3. Nor indicates normoxia, Hyp indicates hypoxia.

The expression of HIF-1α was linked to the abnormal proliferation of pulmonary arterial cells and further influenced the metabolism of mitochondria [27]. We detected the effect of *rutin* on the expression of HIF-1α in both PASMCs and PAECs. Consistent with the PCNA expression, the expression of HIF-1α was enhanced by hypoxia (Figure 6A), the upregulation was inhibited by *rutin*. Scavenged ROS by NAC attenuated *rutin*’s effect. These data suggested that *rutin* inhibited the expression of HIF-1α by reducing the production of ROS.

### 2.6. Discussion

In this study, we mainly highlighted two new concepts from our results. First, *rutin* behaved as an inhibitor of abnormal PA cells proliferation induced by hypoxia by reducing the production of ROS. The main sources of ROS include the mitochondria respiratory chain and NADPH oxidase. Second, the effect of *rutin* on bovine pulmonary artery cells proliferation was attributed to impairment in the NOX_4_-HIF-1α pathway.

As reported, hypoxia increased the production of ROS and regulated PA cells proliferation, which is a key component of pulmonary vascular remodeling, leading to pulmonary hypertension [19,28]. *Rutin* is an antioxidant with many interesting pharmacological effects, such as strengthening and modulating the permeability of the walls of blood vessels [29,30,31]. However, the role of *rutin* in reversing the hypoxia-induced abnormal proliferation by reducing ROS still remained elusive; we hypothesized that the effect of *rutin* on PA cells is relevant to ROS, which might reverse the influence of hypoxia on ROS. In order to test our hypothesis, we used immunofluorescence with CM-H_2_DCFDA or mitosox to evaluate the production and cell location of ROS under hypoxia. Consistent with previous reports, our data showed hypoxia increased ROS generation, and *rutin* can block this effect, which verified our concepts. The main sources of ROS regulated by *rutin* under hypoxic circumstance were the mitochondria respiratory chain and NADPH oxidase. These observations indicated that mtROS contributed to the effect of *rutin* on PAECs under hypoxia.

It is well known that the NADPH (nicotinamideadenine dinucleotide phosphate) oxidase family includes seven isoforms. Nox4 is a unique Nox isoform which is described as an oxygen sensor [28,32]. Recent studies using Nox4 transgenic and knockout mouse models, show a new paradigm is emerging that Nox4 may be vasculoprotective and upregulation of this Nox isoform may have potential therapeutic benefit in preventing vascular disease [33]. Based on our previous work, we tried to observe the effect of *rutin* on Nox4 expression and explored the cellular mechanism of this process. Western blot results showed the expression of Nox4 was increased by hypoxia and this effect could be blocked by *rutin*. These observations demonstrated that *rutin* decreased the activity of Nox4, reversed the abnormal increase in ROS production induced by hypoxic circumstance, and subsequently contributed to the potent protecting effect during the pathologic process of hypoxia-induced PAH.

Consistent with previous reports [19,31], our data indicated hypoxia stimulated cell cyclins, increased α-tubulin polymerization in the nucleus, upregulated expression of PCNA (the proliferating cell nuclear antigen), and led to cell proliferation. The effect of hypoxia on PA cells was blocked by *rutin* by a ROS-dependent way. These observations elucidated the molecular mechanisms by which *rutin* modulated the production of ROS, which may lead to insights into defining the role of *rutin* in PA cell proliferation occurring in hypoxic pulmonary remodeling.

In mammals, the primary transcriptional response to hypoxic stress was mediated by the activation of HIF-1α [19,27]. In this study, we found the overexpression of HIF-1α under hypoxia was alleviated by *rutin*, implying that *rutin* may benefit the proliferation of pulmonary artery cells through inhibited HIF-1α activity.

## 3. Experimental Section

### 3.1. Materials

*Rutin* was obtained from the Chinese National Institutes of Food and Drug Control through common commercial sources. NAC (*N*-acetyl-l-cysteine), APO (apocynin), and RE (rotenone) were purchased from Sigma-Aldrich Corporation (St. Louis, MO, USA). Antibodies against Nox4, PCNA, HIF-1α and α-tubulin were purchased from Santa Cruz Biotechnology Inc. (Santa Cruz, Texas, TX, USA). Reactive Oxygen Species (ROS) detection reagents CM-H_2_DCFDA and mitosox red mitochondrial superoxide indicator were obtained from Life Technologies (AB & Invitrogen, Carlsbad, CA, USA). All other reagents were also from common commercial sources.

### 3.2. Cell Isolation and Culture

PAECs and PASMCs were prepared from pulmonary arterial of calf lungs, obtained from a local slaughterhouse and approved by the Harbin Medical University Ethical Committee of Laboratory Animals. The arteries were gently slit open and the innermost layer scraped with a surgical blade to obtain the endothelial cells, after which the arteries were cut into small pieces and the smooth muscle layer affixed tightly to the culture dish. The arterial fractions were then covered with Dulbecco’s modified eagle’s medium (DMEM) with 20% fetal bovine serum (FBS). After the PASMCs were taken out the tissue fractions were then lifted out. The purity and identity of the PAECs and PASMCs were confirmed by anti-CD31 (Santa Cruz), and anti-α-actin, respectively.

### 3.3. Immunofluorescence

After pre-treatment with the ROS inhibitor (apocynin or rotenone) for half an hour, the cells were treated with *rutin* for 2 h, then washed three times with the indicated buffer solution, before incubation with ROS detection reagents or mitosox for the appropriate time. Afterwards, the cells were washed with buffer solution three times as per instructions. The fluorescence intensity was photographed and analyzed by confocal laser scanning microscope (CLSM).

### 3.4. Scratch-Wound Assay

ECs were cultivated in six-well culture plates and scratched with pipette tips. Then the cells were added with *rutin* or NAC plus *rutin* in 5% FBS of DMEM under hypoxia at the indicated concentrations. The same field was photographed at 0 h and 8 h. The area of migration was calculated by counting the migration using Image Pro-Plus 6.0.

### 3.5. Immunocytochemistry

PASMCs were cultured on a poly-l-lysine-coated cover glass (15 mm diameter) and washed with buffer solution, followed by fixation with 4% paraformaldehyde at room temperature for 15 min. After permeabilization with 0.01% Triton X-100 for 10 min, the cells were blocked with 3% normal bovine serum at 37 °C for 30 min, then incubated with anti-α-tubulin primary antibodies (1:50) in PBS at 4 °C overnight. Washed with PBS three times, the cells were incubated with FITC-conjugated secondary antibody (1:100) and Hoechst at 37 °C for 2 h. The images were captured and analyzed by CLSM.

### 3.6. Western Blot Analysis

After pre-treatment with *rutin* (0.1 μmol/L), or NAC (5 μmol/L) plus *rutin* in DMEM with 5% FBS for 24 h, the cells were washed with PBS buffer three times. The lysates were prepared with RIPA lysis buffer. The protein concentration was measured by the bicinchoninic acid protein assay (Pierce, Rockford, IL, USA), based on bovine serum albumin (BSA) standard. The proteins were separated by 10% SDS-PAGE, and electro transferred to a nitrocellulose membrane (Merck Millipore, Beijing, China). Membranes were blocked with 5% milk in TBST for 3 h at 4 °C, followed by incubating with primary antibodies against Nox4, PCNA, HIF-1α (all 1:200 in 5% BSA) at 4 °C overnight. The treated membranes were then exposed to horseradish peroxidase-conjugated secondary antibody (1:10,000, Santa Cruz Biotechnology) for half an hour and enhanced with chemiluminescence reagents.

### 3.7. MTT Assay

PASMCs were cultured in 96-well culture plates before exposing to different reagents at the indicated concentrations for 24 h. After adding 0.5% 3-(4,5-dimethylthiazol-2-yl)-2,5-diphenyl-tetrazolium bromide (MTT), the survival cells formed a blue formazan dye derived from MTT. The reaction was terminated by DMSO and evaluated by spectrophotometric absorbance at 540 nm.

### 3.8. Flow Cytometry

After treating with different reagents at normal concentrations for 24 h, the cultured cells were then washed with PBS, and collected before being fixed with 75% ethanol for another 24 h at 4 °C. After incubation in 0.5 mL PBS (10 μg/mL RnaseA and 100 μg/mL PI) for 30 min at 37 °C away from light, the immobilized cell samples were then measured for DNA fluorescence using a BD FACSCalibur Flow Cytometer (Bedford, MA, USA). The DNA content at each phase of the cell cycle was recorded.

### 3.9. Statistics

Data are presented as mean ± SD. For comparisons between two experimental groups, the student’s paired t-test was used. Significant level was set at *p* < 0.05. Statistical analysis was performed by Statistical Product and Service Solutions version 13.0 (IBM SPSS, Beijing, China).

## 4. Conclusions

We hypothesized that the natural product, *rutin,* was capable of regulating the unusual expression of mitochondrial Nox4 induced by hypoxia and influencing the production of ROS, which subsequently affects the proliferation and remodeling of the pulmonary artery; our observations then provided evidence to support our hypothesis.

In conclusion, *rutin* possesses specific properties in regulating abnormal changes during the pathological process and could serve as a new target in the treatment of hypoxic pulmonary hypertension. Further studies are required to explore the mechanism of the cell signaling pathways with regard to the antioxidant mechanism process of *rutin* on hypoxia-induced pulmonary arterial cell proliferation.

## References

[1] Parent F., Bachir D., Inamo J., Lionnet F., Driss F., Loko G., Habibi A., Bennani S., Savale L., Adnot S. (2011). A hemodynamic study of pulmonary hypertension in sickle cell disease. N. Engl. J. Med..

[2] Tabima D.M., Frizzell S., Gladwin M.T. (2012). Reactive oxygen and nitrogen species in pulmonary hypertension. Free Radic. Biol. Med..

[3] Voelkel N.F., Tuder R.M. (2000). Hypoxia-induced pulmonary vascular remodeling: A model for what human disease?. J. Clin. Invest..

[4] Stenmark K.R., Meyrick B., Galie N., Mooi W.J., McMurtry I.F. (2009). Animal models of pulmonary arterial hypertension: The hope for etiological discovery and pharmacological cure. Am. J. Physiol. Lung Cell. Mol. Physiol..

[5] Mandegar M., Fung Y.C., Huang W., Remillard C.V., Rubin L.J., Yuan J.X. (2004). Cellular and molecular mechanisms of pulmonary vascular remodeling: Role in the development of pulmonary hypertension. Microvasc. Res..

[6] Morrell N.W., Adnot S., Archer S.L., Dupuis J., Jones P.L., MacLean M.R., McMurtry I.F., Stenmark K.R., Thistlethwaite P.A., Weissmann N. (2009). Cellular and molecular basis of pulmonary arterial hypertension. J. Am. Coll. Cardiol..

[7] Ogura S., Shimosawa T., Mu S., Sonobe T., Kawakami-Mori F., Wang H., Uetake Y., Yoshida K., Yatomi Y., Shirai M. (2013). Oxidative stress augments pulmonary hypertension in chronically hypoxic mice overexpressing the oxidized LDL receptor. Am. J. Physiol. Heart Circ. Physiol..

[8] Fan Y.F., Zhang R., Jiang X., Wen L., Wu D.C., Liu D., Yuan P., Wang Y.L., Jing Z.C. (2013). The phosphodiesterase-5 inhibitor vardenafil reduces oxidative stress while reversing pulmonary arterial hypertension. Cardiovasc. Res..

[9] Konduri G.G., Bakhutashvili I., Eis A., Pritchard K. (2007). Oxidant stress from uncoupled nitric oxide synthase impairs vasodilation in fetal lambs with persistent pulmonary hypertension. Am. J. Physiol. Heart Circ. Physiol..

[10] Waypa G.B., Marks J.D., Mack M.M., Boriboun C., Mungai P.T., Schumacker P.T. (2002). Mitochondrial reactive oxygen species trigger calcium increases during hypoxia in pulmonary arterial myocytes. Circ. Res..

[11] Wedgwood S., Dettman R.W., Black S.M. (2001). ET-1 stimulates pulmonary arterial smooth muscle cell proliferation via induction of reactive oxygen species. Am. J. Physiol. Lung Cell. Mol. Physiol..

[12] Ray P.D., Huang B.W., Tsuji Y. (2012). Reactive oxygen species (ROS) homeostasis and redox regulation in cellular signaling. Cell. Signal..

[13] Basuroy S., Tcheranova D., Bhattacharya S., Leffler C.W., Parfenova H. (2011). Nox4 NADPH oxidase-derived reactive oxygen species, via endogenous carbon monoxide, promote survival of brain endothelial cells during TNF-α-induced apoptosis. Am. J. Physiol. Cell Physiol..

[14] Kuhr F.K., Smith K.A., Song M.Y., Levitan I., Yuan J.X. (2012). New mechanisms of pulmonary arterial hypertension: Role of Ca^2+^ signaling. Am. J. Physiol. Heart Circ. Physiol..

[15] Shimoda L.A., Laurie S.S. (2013). Vascular remodeling in pulmonary hypertension. J. Mol. Med..

[16] Kelman Z. (1997). PCNA: Structure, functions and interactions. Oncogene.

[17] Strzalka W., Ziemienowicz A. (2011). Proliferating cell nuclear antigen (PCNA): A key factor in DNA replication and cell cycle regulation. Ann. Bot..

[18] Zhang D., Ma C., Li S., Ran Y., Chen J., Lu P., Shi S., Zhu D. (2012). Effect of Mitofusin 2 on smooth muscle cells proliferation in hypoxic pulmonary hypertension. Microvasc. Res..

[19] Semenza G.L. (2012). Hypoxia-inducible factors in physiology and medicine. Cell.

[20] Shimoda L.A., Semenza G.L. (2011). HIF and the lung: Role of hypoxia-inducible factors in pulmonary development and disease. Am. J. Respir. Crit. Care Med..

[21] La Casa C., Villegas I., Alarcón de la Lastra C., Motilva V., Martin Calero M.J. (2000). Evidence for protective and antioxidant properties of *rutin*, a natural flavone, against ethanol induced gastric lesions. J. Ethnopharmacol..

[22] Kamalakkannan N., Prince P.S. (2006). Antihyperglycaemic and antioxidant effect of *rutin*, a polyphenolic flavonoid, in streptozotocin-induced diabetic wistar rats. Basic Clin. Pharmacol. Toxicol..

[23] Li Q., Niu S., Wang R., Li Y., Zhang R., Zhu D. (2012). Mechanisms that underlie the induction of vasodilation in pulmonary artery by *rutin*. Int. Angiol..

[24] Xiao G.F., Xu S.H., Chao Y., Xie L.D., Xu C.S., Wang H.J. (2014). PPARdelta activation inhibits homocysteine-induced p22(phox) expression in EA.hy926 cells through reactive oxygen species/p38MAPK pathway. Eur. J. Pharmacol..

[25] Wedgwood S., Lakshminrusimha S., Czech L., Schumacker P.T., Steinhorn R.H. (2013). Increased p22(phox)/Nox4 expression is involved in remodeling through hydrogen peroxide signaling in experimental persistent pulmonary hypertension of the newborn. Antioxid. Redox Signal..

[26] Stones R., Benoist D., Peckham M., White E. (2013). Microtubule proliferation in right ventricular myocytes of rats with monocrotaline-induced pulmonary hypertension. J. Mol. Cell. Cardiol..

[27] Wolin M.S. (2012). Novel role for the regulation of mitochondrial fission by hypoxia inducible factor-1α in the control of smooth muscle remodeling and progression of pulmonary hypertension. Circ. Res..

[28] Yang X., Sheares K.K., Davie N., Upton P.D., Taylor G.W., Horsley J., Wharton J., Morrell N.W. (2002). Hypoxic induction of cox-2 regulates proliferation of human pulmonary artery smooth muscle cells. Am. J. Respir. Cell Mol. Biol..

[29] Khan R.A., Khan M.R., Sahreen S. (2012). Protective effects of *rutin* against potassium bromate induced nephrotoxicity in rats. BMC Complement. Altern. Med..

[30] Azevedo M.I., Pereira A.F., Nogueira R.B., Rolim F.E., Brito G.A., Wong D.V., Lima-Junior R.C., de Albuquerque Ribeiro R., Vale M.L. (2013). The antioxidant effects of the flavonoids *rutin* and quercetin inhibit oxaliplatin-induced chronic painful peripheral neuropathy. Mol. Pain.

[31] Panchal S.K., Poudyal H., Arumugam T.V., Brown L. (2011). *Rutin* attenuates metabolic changes, nonalcoholic steatohepatitis, and cardiovascular remodeling in high-carbohydrate, high-fat diet-fed rats. J. Nutr..

[32] Montezano A.C., Burger D., Ceravolo G.S., Yusuf H., Montero M., Touyz R.M. (2011). Novel Nox homologues in the vasculature: Focusing on Nox4 and Nox5. Clin. Sci..

[33] Schröder K., Zhang M., Benkhoff S., Mieth A., Pliquett R., Kosowski J., Kruse C., Lüdike P., Michaelis U.R., Weissmann N. (2012). Nox4 is a protective reactive oxygen species generating vascular NADPH oxidase. Circ. Res..

